# Exploring the impact of a Tele-health intervention on specific quality of life domains and psychological well-being in diabetic patients within the Whole Systems Demonstrator Questionnaire Study

**Published:** 2012-06-15

**Authors:** Shashivadan Hirani, Lorna Rixon, Martin Cartwright, Michelle Beynon, Helen Doll, Stanton Newman

**Affiliations:** School of Health Sciences, City University, UK; School of Health Sciences, City University, UK; School of Health Sciences, City University, UK; School of Health Sciences, City University, UK; University of East Anglia, UK; School of Health Sciences, City University, UK

**Keywords:** telehealth, diabetes, quality of life, psychological outcomes, Whole System Demonstrator

## Abstract

**Introduction:**

Much is written about the promise of Telehealth (TH) and there is great enthusiasm about its potential. However, many studies of TH do not meet orthodox quality standards. Evidence-based on assimilating findings from a few small trials of variable methodological quality presents equivocal findings and makes results difficult to interpret. Robust evidence to inform policy decisions is considered to be lacking (Ekeland et al., 2010). This remains the case when specific long-term conditions such as diabetes are considered with regards to health related quality of life (HRQoL) outcomes. The WSD study aimed to address some of these concerns.

**Aims:**

This study investigated the impact of TH on disease-specific HRQoL, generic HRQoL and psychological outcomes (anxiety and depression) in patients with diabetes, utilising longitudinal questionnaire data from the WSD Telehealth Study.

**Design:**

The WSD Telehealth Questionnaire Study is pragmatic cluster-randomised controlled trial evaluating a broad range of patient-reported outcome measures. Participants were recruited from three sites in the UK (Cornwall, Kent and Newham). The current analyses focus on participants with diabetes.

**Methods:**

Over 400 participants with diabetes completed measures of disease-specific HRQoL (DHP), generic HRQoL (UK SF-12; EQ5D), anxiety (Brief STAI) and depression (CESD-10) at baseline. Short- and long-term follow-up assessments were conducted. Multi-level modelling analyses of difference in outcomes between trial arm groups and across time were conducted with appropriate covariates.

**Results and Conclusions:**

Findings and conclusions from the WSD Telehealth Study are embargoed until these analyses have been peer-reviewed and accepted for publication.

